# An empirical evaluation of the impact of missing data on treatment effect

**DOI:** 10.1186/1745-6215-16-S2-P136

**Published:** 2015-11-16

**Authors:** Royes Joseph, Julius Sim, Reuben Ogollah, Martyn Lewis

**Affiliations:** 1 Research Institute for Primary Care and Health Sciences, Keele University, Keele, Staffordshire, UK

## Objectives

Missing data represent a potential source of bias in randomized clinical trials (RCTs). A simple approach that makes use of the responses subsequently obtained via reminder is proposed to assess the validity of the inferences from a missing at random (MAR)-based primary analysis of incomplete RCTs.

## Methods

We explored mechanism behind the reminder responses in two pragmatic RCTs - the TATE and STarT Back trials - by utilizing the fact that data that are recovered through reminders would otherwise have been missing. The present approach considered two data scenarios: (i) with the actual dataset and (ii) with a modified dataset, where outcome responses obtained after a certain number of reminders were treated as missing. The impact of the reminder responses was assessed by comparing the estimates from MAR-based analyses between the two data scenarios.

## Results

In the TATE trial, the reminder approach showed that an MAR-based analysis was likely to yield biased estimates of treatment effect. Therefore, further sensitivity analyses were required under a range of plausible missing not at random (MNAR) assumptions. However, in the STarT Back trial, this approach showed that an MAR-based analysis was likely to yield an unbiased estimate of treatment effect.

## Conclusion

The proposed reminder approach can be used to assess the robustness of the MAR assumption by checking expected consistency in MAR-based estimates. If the results deviate, then MAR-based estimates are likely to be biased, and analyses incorporating a range of plausible MNAR assumptions are advisable at least as sensitivity tests for the evaluation of treatment effect.

